# Inequities in maternal health services utilization in Ethiopia 2000–2016: magnitude, trends, and determinants

**DOI:** 10.1186/s12978-018-0556-x

**Published:** 2018-07-04

**Authors:** Emebet Gebre, Alemayehu Worku, Fawole Bukola

**Affiliations:** 1 Department of Obstetrics and Gynaecology, College of Medicine, Pan Africa University Life and Earth Sciences Institute, University of Ibadan, Ibadan, Nigeria; 2 School of Public Health, Addis Ababa University, Addis Ababa, Ethiopia

**Keywords:** Determinants, Inequities, Maternal health services, Trend, Utilization

## Abstract

**Background:**

Inequities in maternal health services utilization constitute a major challenge in maternal mortality reduction in Ethiopia. We sought to assess magnitude, trends, and determinants of inequities in maternal health services utilization in Ethiopia from 2000 to 2016.

**Methods:**

The study utilized data from the 2000 and 2016 Ethiopia Demographic and Health Surveys, which were done based on a cross sectional survey design. The wealth-related inequities were assessed by concentration curve and horizontal inequity indices. Trends in inequities were assessed by comparing the concentration indices of maternal health services utilization variables between the 2000 and 2016 surveys using Wagstaff two groups concentration indices comparison method. Finally, the inequities were decomposed into its contributing factors using Wagstaff method of analysis.

**Results:**

Wealth-related inequities were significantly high in 2016: with horizontal inequities indices and residual regression error of antenatal care, skilled birth attendance, and postnatal care service utilization (− 0.09 and − 0.01), (− 0.06 and 0.01), and (− 0.11 and 0.0001), respectively. These indices increased significantly in 2016 when it is compared with the 2000 indices’ with the respective concentration indices difference of − 0.05, 0.05, and − 0.07. The related all *p*-values were < 0.0001. The main determinants of inequities were low-economic status, illiteracy, rural residence, no occupation, and fewer accesses to mass media.

**Conclusions:**

In Ethiopia, maternal health services utilization inequities were significantly high and increased in 2016 compared to 2000. Women who are poor, rural resident, uneducated, unemployed, and fewer mass media exposed are the most disadvantaged. Targeting maternal health interventions for the underserved women is essential to reduce maternal mortality in the country.

**Electronic supplementary material:**

The online version of this article (10.1186/s12978-018-0556-x) contains supplementary material, which is available to authorized users.

## Plain English summary

Unfair difference among population groups (inequities) in maternal health services utilization is one of the challenges in reduction of maternal mortality in Ethiopia. The aims of the study were to assess magnitude, trends, and determinants of inequities in maternal health services utilization in Ethiopia from 2000 to 2016. Maternal health services utilization inequities were significantly high and increased in 2016 compared to 2000 with low-economic status, illiteracy, rural residence, no occupation, and lack of access to mass media being the main determinants. In Ethiopia, the rich women were more likely to utilize maternal health services than the poor. The most disadvantaged women were the poor, rural resident, uneducated, unemployed, and with fewer/no mass media access. Targeting maternal health interventions for the underserved women is essential to reduce maternal mortality in the country.

## Background

Worldwide 800 women die each day from childbirth and pregnancy related complications [1]. Out of the total worldwide maternal deaths, 99% occurred in developing countries and sub-Saharan Africa bears the largest share (66%). Relative to other developing countries, maternal mortality^1^ remains high in Ethiopia with a maternal mortality ratio of 412 per 100, 000 live birth [2].

Inequities^2^ in maternal health services utilization in Ethiopia is one of the highest in the world [3], which is also one of the contributing factors for high maternal mortality in the country [4], although developing an equitable standard of health services for all segments of the population is one of the general health policies of Ethiopian government [5].

Maternal and new-born deaths can be prevented by implementing key.

essential services for mothers including antenatal care (having at least four or more antenatal care visits during pregnancy), skilled attendance at birth, and postnatal care [6, 7].

In many developing countries including Ethiopia, access to these lifesaving services is limited among key population groups due to low socioeconomic status [8–14] and remains as one of the challenges in maternal mortality reduction [15]. Thus understanding the current status, trend, and contributing factors of inequities in maternal health service utilization is crucial to achieve the Sustainable Development Goal (SDG) that aims to reduce maternal mortality to 70 per 100,000 live births by 2030 [16]. Inequities in maternal health services utilization has been reported in Ethiopia [9–14], although the approaches were divergent. Some of these studies were based on a subset of population that were on the two extreme poles of wealth continuum between the richest and the poorest segment of the population [17]. This approach excludes the subset of population between these two poles and thus does not provide a full picture of the effect of wealth index on maternal health services utilisation [18]. Utilisation of health services and health inequalities do not depend only on a wealth factor but also other need factors that increase individual’s attendance of health care can confound the effect of wealth index on maternal health services utilisation. The effect of such need factors was not taken into account in some studies [11]. Studies that include all the population segments irrespective of wealth status of individuals as well as those that account for the effect of potential confounders (need factors) are required to fully understand health inequalities and its determinants [18, 19]. Measurement of inequity and its trends as data becomes available is important to monitor regularly the country progress towards achieving sustainable development goal (achieving universal health coverage for essential maternal health interventions for all by 2030) [16]. Thus, the aims of this study were to assess the magnitude, trends, and determinants of inequities in maternal health services utilization in Ethiopia using nationally representative data that became available recently. Such that, the country’s progress towards achieving sustainable development goals can be tracked.

## Methods

### Data

We used nationally representative Ethiopia Demographic and Health Surveys (EDHS) data from 2000 to 2016 publicly available via Measure DHS. EDHS are nationally representative household surveys conducted at 5-yearly intervals with a strong focus on indicators of maternal and child health, reproductive health, fertility, nutrition, mortality, and self-reported health behaviors among adults. Demographic and health surveys are considered as providing an important source of monitoring population health indicators and vital statistics in middle and low-income countries and known by its design, which are highly comparable among different settings and over time [20, 21]. All EDHS were conducted using a similar approach in sample design, sample selection, and survey methodology (each round survey methodology is stated in the respective reports) and ethically approved [2, 22–25].

### Socioeconomic rank

We used the 2016 EDHS wealth index (WI) variable as a living standard measure for the 2016 data. Since WI variable was not available in the 2000 EDHS data, we constructed the WI variable using the Principal Component Analysis (PCA) as a measure of socioeconomic status used in DHS reports of many countries [26]. The variables included in the PCA were durable assets ownership: radio, car, refrigerator, television, motorcycle, and bicycle; housing characteristics: number of rooms for sleeping and building material (wall, floor and roof); and access to utilities and infrastructure: electricity supply, source of drinking water, and sanitation facility.

### Measures

We assessed inequities in antenatal care (ANC), skilled birth attendant (SBA) and postnatal care (PNC) services utilization – the most prioritized maternal health interventions in securing the continuum of care for maternal and child health [27]. ANC was calculated as the number of women who had at least four or more ANC visits for the last birth in five years preceding the survey. Similarly, SBA was defined as the number of women who were assisted by a skilled health provider (Doctor or Nurse or Midwife for the 2000 EDHS data and Doctor or Nurse or Midwife or Health Officer or Health Extension Worker for 2016 EDHS data) for the last live birth in five years preceding the survey. PNC was computed as the number of women who had postnatal check-up within two days after delivery for the last birth in five years preceding the survey except for the 2016 surveys (within two years preceding the survey).

### Explanatory variables descriptions

The explanatory variables were women’s wealth status, residence (urban or rural), education, occupation, mass media exposure, respondent’s current age and current marital status.

### Data analysis

#### Measuring inequity

Inequities in Maternal Health Services (MHS) utilization were measured by Horizontal Inequity Indices (HII) and concentration curve following Wagstaff method of analysis [28]. Since age and four or more birth order of a woman may affect wealth status of a woman [29] and may correlate with MHS utilization [30]; inequities were determined after standardizing MHS utilization variables using indirect approach. To indirectly standardize each of the health variables, a nonlinear method of estimation using logit model was used.

1 $$ {y}_i=\alpha +\sum \limits_j{\beta}_j{\chi}_{ji}+\sum \limits_k{y}_k{z}_{ki}+{\varepsilon}_{i,} $$

where α*,* β*,* and γ are parameter vectors; *y*_*i*_ is MHS utilization; *x* is the need factors (confounding variables) such as women age and four or more birth order; *z* is non-need factors (non-confounding variables) to control or estimate the partial correlation with confounding variables. The non-need factors were education status, rural resident, low-economy status, current marital status, occupation, mass media exposure (a predictor of MHS utilization [31]). Ɛ is an error term. For our case we used Logit model.

The indirectly standardized MHS utilization estimate is given by the difference between actual MHS utilization y_i_ and the expected utilization (utilization expected only from need factors), plus the total sample mean of MHS utilization $\overline{y}$ [32].

2 $$ {\widehat{y}}_i^{IS}={y}_i-{\widehat{y}}_i^X+\overline{y} $$

In this study, concentration curve plots the cumulative percentage of the MHS utilization variable (on the vertical axis) against the cumulative percentage of the population ranked by wealth index starting with the poorest (left) and ending with the better-off (right) on the horizontal axis [33, 34].

Concentration Index (CI) is calculated from concentration curve as it is defined as twice the area between the concentration curve and the line of equality [34]. Using eq. 3 [35].

3 $$ C=\frac{2}{n\cdot \mu }{\sum}_{i=1}^n{y}_i{R}_i-1, $$

where C is the concentration index, *μ* is the mean of *y*_*i*_ (MHS utilization), *R*_*i*_ is the fractional rank of the *i*th individual in the income distribution.

Concentration Index can also be calculated simply by following the “convenient regression approach”. This kind of calculation gives both the estimates and also the standard errors to produce statistical inferences.

It can be written as follows:

4 $$ 2{\sigma}_r^2\left(y{}_i/\mu \right)=\alpha +\beta {r}_i+{\varepsilon}_i $$

Where, *β* is an estimated concentration index, ${\sigma}_r^2$ is the variance of the rank (r), the other variables are as defined in eq. (3), and Ɛ is the stochastic error term.

Then, we got MHS Horizontal Inequity Indices (HII) and residual by calculating the concentration indices using the need standardized MHS utilization variables [36]. When there is no inequality, the horizontal inequity index will be zero and the concentration curve lies on the diagonal line starting from the origin (line of equality); which means all individuals, regardless of their economic status have the same value of the health variable. If there is inequity, CI will be negative or positive indicating that the MHS utilization variable is more concentrated among poor or better-off group of people and concentration curve lies above or below the line of equality and the HII value lies between − 1 and 1. The further concentration curve lays from the line of equality, the greater the degree of inequality in MHS utilization across income groups. Similarly, the higher the absolute CI value is the greater the inequity in MHS utilization [28, 32].

Trends in inequities of MHS utilization were investigated using Wagstaff two groups (2000 and 2016 EDHS) concentration indices comparison method via the “CONINDEX” STATA commands [37].

### Decomposition

To examine the socioeconomic determinants of inequities in MHS utilization, decomposition of the indirectly standardized concentration indices were done following a method of analysis proposed by Wagstaff et al. [28, 36].

A linear regression model joining our variables of interest (MHS utilization), y, to a set of *k* determining factors, x_k_:

5 $$ {y}_i=\alpha +\sum \limits_k{\beta}_k{x}_{ki}+{\varepsilon}_i, $$

Where, β_k_ is coefficient of health determinants, Ɛ_i_ is an error term.

Given the association between *yi* and *x*_*ki*_ in Eq. (5), the concentration index for *y*, *CI*, can be written as:

6 $$ c=\sum \limits_k\left(\frac{\beta_k{\overline{x}}_k}{\mu}\right)\kern0.5em {c}_k+\frac{GC_{\varepsilon }}{\mu }={c}_{\widehat{y}}+\frac{GC_{\varepsilon }}{\mu }, $$

where, β_k_ is the regression coefficient of variable x_k_, μ is the mean of y, ${\overline{x}}_k$ is the mean of variable x_k_, C_k_ is CI of variable x_k,_ μ is the mean of MHS utilization variable and GC_Ɛ_ is generalized CI for the an error term (Ɛ); which can be computed as a residual and can be defined as:

7 $$ {GC}_{\varepsilon }=\frac{2}{n}{\sum}_{i=1}^n{\varepsilon}_i{R}_1, $$

The CI in eq. (6) shows that it is made up of two components. The first component is deterministic (explained) which is equal to weighted sum of concentration indices of *k* regressors, where the weight or “share” for x_k_ is simply the elasticity of y with respect to x_k_ when evaluated at the sample mean. An elasticity is a unit-free measure of (partial) association, i.e. the percentage change in the dependent variable (MHS utilization in this case) associated with a percentage change in the explanatory variable of y with respect to each x_k, (_$\frac{\beta_k{\overline{x}}_k}{\mu }$). But, the second component is the residual, which reflects the inequality in health service utilization that cannot be explained by systematic variation in the x_*k*_ across socioeconomic groups [28, 32].

The data were processed and analyzed using EXCEL and STATA version 13.0 [34]. In addition, ADePT software version 6 was used to analyse the socioeconomic inequities and decomposition [38]. We incorporated EDHS data unequal sampling weight and household clustering effect in the analyses [39]. Sample-weighted data were used for all of the analyses to adjust for the under-sampling, the over-sampling, and the response rates differences in different regions.

## Results

### Demographic and socio-economic characteristics of respondents

The study participants in Ethiopia DHS were taken from the nine geographic regions and two administrative states (Addis Ababa City administration and Dire Dawa city council). The proportion of population that resides in rural area in each of these nine regions ranges from 50% in Harari region to 90% in Southern Nation Nationality People Region (SNNPR). Majority of the respondents were 15–19 years old, rural resident, illiterate, married, and from Oromya region both in 2000 and 2016 (Table 1).

**Table 1:** Demographic and socio-economic characteristics of respondents in Ethiopia by survey year (2000 & 2016)

Characteristics		Year	
		2000 N (%)	2016 N (%)
Age (years)	15–19	3584(23.3)	3498(22.3)
	20–24	2844 (18.5)	2903(18.5)
	25–29	2716 (17.7)	2845(18.1)
	30–34	1902(12.4)	2241(14.3)
	35–39	1762(11.5)	1917(12.2)
	40–44	1324 (8.6)	1302(8.3)
	45–49	1235 (8.0)	977(6.2)
Residence	Urban	4543(29.6)	5348(34.1)
	Rural	10,824(70.4)	10,335(65.9)
Education Level	No education	10,586 (68.9)	7033(44.8)
	Primary	2530(16.5)	5213 (33.2)
	Secondary	2092(13.6)	2238 (14.3)
	Higher	159(1.0)	1199(7.7)
Wealth index	Poorest	2885(21.1)	3894(24.8)
	Poorer	806(5.9)	2046(13.1)
	Medium	2731(19.9)	2002(12.8)
	Rich	2553(18.7)	2042(13.0)
	Richest	4684(34.3)	5699(36.3)
Marital status	Never married	3979 (25.9)	4278(27.3)
	Married	9203(59.9)	9602 (61.2)
	Living together	177(1.2)	222 (1.4)
	Widowed	657(4.3)	451 (2.9)
	Divorced	926(6.0)	878(5.6)
	Not living together	425(2.8)	252(1.6)
Region	Tigray	1306(8.5)	1682(10.7)
	Affar	858 (6.6)	1128(7.2)
	Amhara	1909(12.4)	1719 (11.0)
	Oromiya	2578(16.8)	1892 (12.1)
	Somali	844(5.5)	1391 (8.9)
	Benshal-gumz	992(6.5)	1126 (7.2)
	SNNPR	2028(13.2)	1849 (11.8)
	Gambela	876 (5.7)	1035 (6.6)
	Harari	908(5.9)	906 (5.8)
	Addis	2015(13.11)	1824(11.6)
	Dire Dawa	1053(6.9)	1131(7.2)

### Inequities in maternal health services (MHS) utilization in Ethiopia in 2016

Inequities in MHS utilization were assessed by concentration curve, concentration indices, and horizontal inequity indices.

**Fig. 1:**
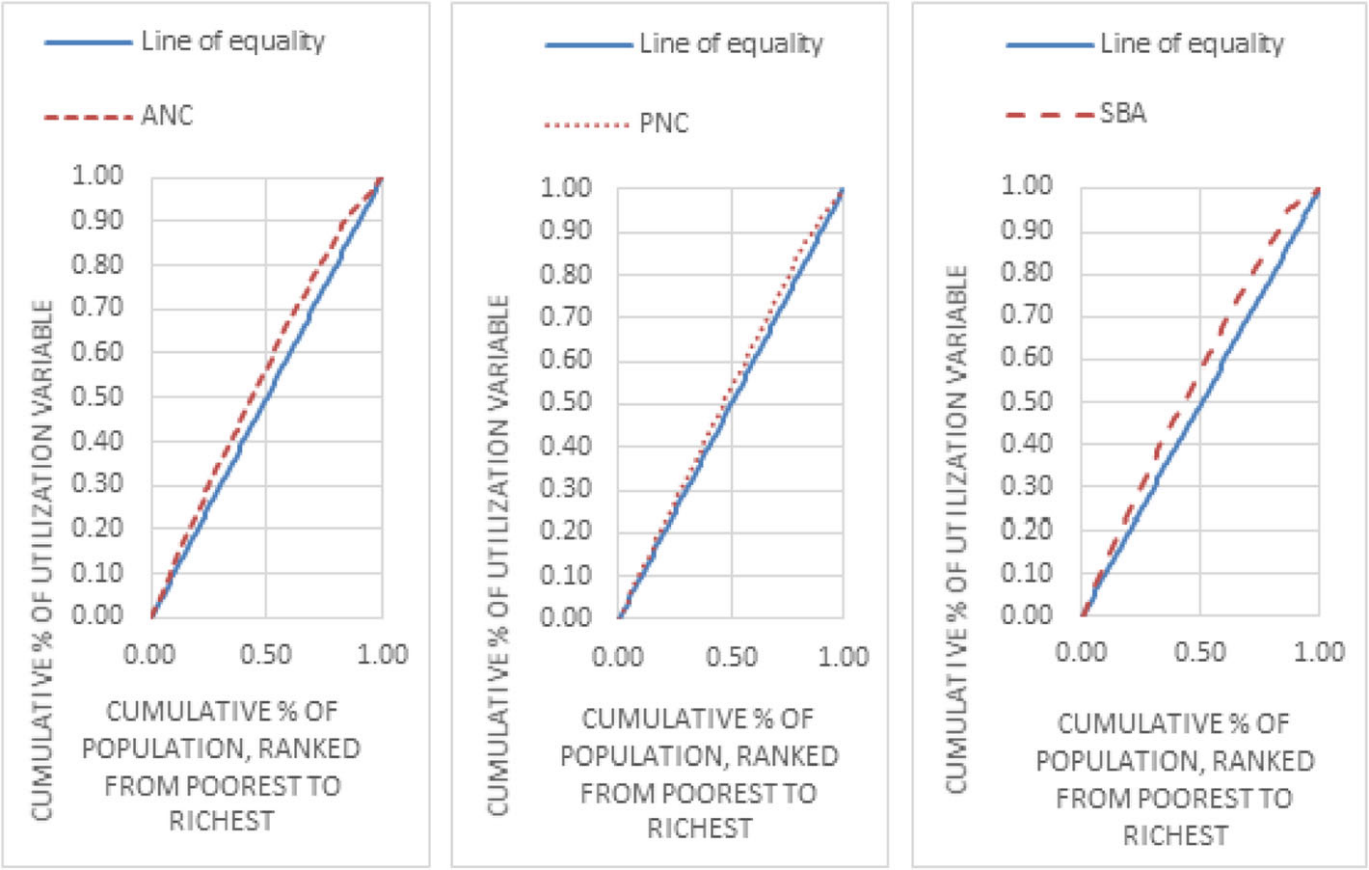
Maternal health services utilization concentration curves in Ethiopia in 2016

Figure 1 shows concentration curves of MHS utilization variables. All of the concentration curves lie above the line of equity, indicating that the non-utilization is concentrated among the poorest than the better-off women.

The horizontal inequity indices (HII) and their residual regression errors of ANC, SBA, and PNC services utilization were found as (HII and residual regression errors) (− 0.10 and 0.01), (− 0.11 and 0.0001), and (− 0.06 and − 0.01), respectively in 2016. Similarly, the horizontal inequity indices show considerable inequity with MHS non- utilization concentrated among the poor women than those who were better-off.

### Trends in MHS utilization inequities by wealth quintile in Ethiopia from 2000 to 2016

The likelihood of better-off than poor women in the utilization of ANC, SBA, and PNC check-up services –inequities– increased significantly in 2016 related to 2016 (Table 2, Additional files 1, 2, and 3).

**Table 2:** The trends of MHS utilization inequities in Ethiopia from 2000 to 2016

					**Z-test**		
		CI	SE	*p*-value	Difference	SE	*p*-value
ANC	2000	− 0.04	0.01	0.0001			
	2016	−0.09	0.01	0.0001			
					−0.0518	0.0099	< 0.0001
PNC	2000	−0.0042	0.0015	0.0060			
	2016	−0. 0540	0.0051	< 0.0001			
					−0.0499	0.0053	< 0.0001
SBA	2000	−0.03	0.00	< 0.0001			
	2016	−0.10	0.01	< 0.0001			
					−0.07	0.01	< 0.0001

### Decomposition of the concentration indices

We decomposed the determinants of MHS utilization inequities in Ethiopia using data from the 2016 EDHS data.

**Fig. 2:**
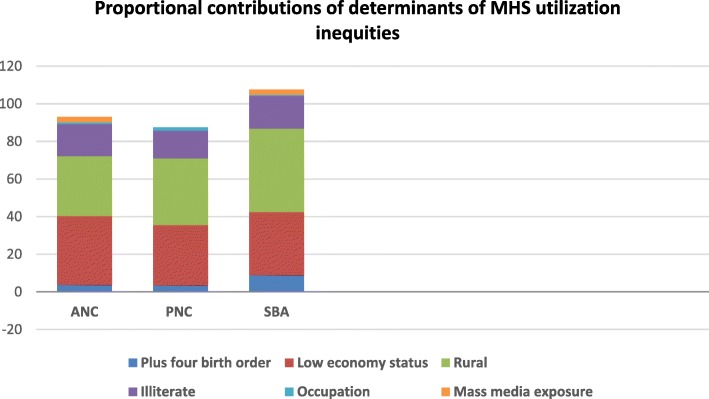
Proportional contributions of determinants of MHS utilization inequities in Ethiopia in 2016

The dominant determinants of inequities in ANC service utilization (three or fewer ANC visits) inequity were low wealth status (37%), followed by rural residence (32%) and being illiterate (17%). Additionally, less than twice a week mass media exposure (3%) was found to be a significant determinant of inequity (Table 3, Fig. 2).

**Table 3:** Decomposition of MHS utilization concentration indices (Ethiopia, 2016)

	Coefficient	Elasiticity	Covariates CI	Absolute Contribution	Percentage Contribution
ANC					
Standardize variables					
Respondent’s current age	−0.003	−0.116	0.001	0.000	0
Birth order 4^+^	0.057	0.045	−0.080	− 0.004	4
Control variables					
Low wealth status	0.091	0.062	−0.564	−0.035	37
Rural	0.235	0.293	−0.104	−0.030	32
Illiterate	0.117	0.112	−0.144	−0.016	17
Occupation	−0.019	−0.015	0.067	−0.001	1
Mass media exposure	0.239	0.445	−0.006	−0.003	3
Current marital status	0.007	0.013	−0.008	0.000	0
Residual					−0.01
PNC					
Standardizing variables					
Respondent’s current age	−0.001	−0.034	0.001	0.000	0
Birth order 4^+^	0.044	0.025	−0.080	−0.002	4
Control variables					
Wealth status (low)	0.055	0.032	−0.564	−0.018	32
Illiterate	0.079	0.058	−0.144	−0.008	15
Rural	0.230	0.191	−0.104	−0.020	35
Mass media exposure	0.012	−0.005	−0.006	0.000	0
Occupation	−0.026	−0.014	0.067	−0.001	2
Current marital status	−0.002	−0.003	− 0.007	0.000	0
Residual					−0.01
SBA					
Standardizing variables					
Respondent’s current age	−0.003	−0.160	0.001	0.000	0
Birth order 4^+^	0.153	0.141	−0.071	−0.010	9
Control variables					
Low wealth status	0.094	0.071	−0.532	−0.038	34
Rural	0.425	0.560	−0.089	−0.050	44
Illiterate	0.149	0.148	−0.132	−0.020	17
Occupation	−0.014	−0.010	0.066	−0.001	1
Mass media exposure	0.173	0.512	−0.006	−0.003	3
Current marital status	−0.001	0.000	−0.005	0.000	0
Residual					0.00

The leading determinants of inequities in SBA service utilization were being a rural resident (44%) followed by low wealth status (34%) and illiteracy (17%). Having less than twice a week mass media exposure (3%) was also found as a significant determinant of the inequity (Table 3, Fig. 2).

The major determinants of inequity in PNC check-up were illiteracy (35%) followed by low wealth status (32%) and rural residence (15%). In addition, occupation (2%) was found as a significant determinant of the inequity (Table 3, Fig. 2).

The decomposition residual regression errors of inequities in ANC, PNC, and SBA services utilization were found as − 0.01, − 0.01, and 0.0001, respectively, which are very small and indicate factors included in the decomposition analysis explained most of the inequities (Table 3).

Generally, considerable inequity exists among the study population poor, rural resident, illiterate, unemployed, and women with no/fewer mass media exposure.

## Discussion

We found that considerable inequities exist among the study population and non-utilization of MHS is concentrated among the disadvantage women. In Ethiopia, poor women were less likely than better-off in utilizing ANC, SBA, and PNC services in 2016. This finding is similar to earlier studies [11, 17, 40–47].

Compared to the baseline, 2000 survey; the likelihood of ANC, SBA, and PNC services utilization was significantly higher among better-off than poor women in 2016. This finding is similar to previous reports [9, 17, 41, 48–50], but contradicts some former studies [47, 50]. This may be for PNC due to PNC service utilization increased significantly among better-off women but not among poor from 2000 to 2016, for SBA due to statistically insignificant change in utilization of SBA service coverage amongst both well off and poorest from 2000 to 2016 [22]; and generally due to the rapid population growth observed in the country from 2000 to 2017 [51], which may increase the health care demands of the people, a low health facility to population ratio, and it may be also due to low health workforce (0.7 health workers per population) in the country compared to the WHO recommendation (2.3 health workers per 1000 population) [52]. However, it should be noted that the computation of wealth indices for the two surveys is different with urban-rural differences taken into account in the 2016 survey.

Low economic status was found as a major determinant of inequities in MHS utilization. This may be poor women may not have money to cover payments for transport and either for the service or other expenses to bring and keep families at a health facility. This is similar to prior reports [41, 53–55].

Similarly, rural residence was found as a major determinant of inequities in all the studied MHS utilization. This finding is similar to some previous studies [11, 42, 44, 55–57]. Lack of access to health care services in rural areas due to inaccessibility of health facilities and professionals, lack of transportation services, and less access to infrastructures and services play a key role in the observed inequality. Thus, rural and remote areas are often underserved by health workers [52]. In addition, women in rural places are less educated and less autonomous than their urban counterparts to actively seek health cares.

Likewise, illiteracy is a major determinant of inequity in MHS utilization. Illiterate women are often unemployed, lack independent decision-making ability, and awareness about the importance of having MHS. A similar finding was reported from Vietnam [54].

Mass media exposure was found to be a significant determinant of inequities in MHS utilization. Women exposed to mass media at least twice a week were more likely to utilize MHS than those less exposed. This finding is similar to a report from India [53, 58]. This may be because those more exposed to mass media may have good awareness about the importance of utilizing the services.

This study does have some limitations. The first limitation is recall bias because the responses were based on the mother’s recall. The second limitation is related to asset indices. We identified that the wealthiest quintile inclined to reside in urban places; this indicates that the wealth inequalities may be related to rural/urban disparities. The third limitation is household asset based inequalities exclude inequalities related to age, ethnic group, or position in the household family structure. The fourth limitation is the absence of some need factors in EDHS for interventions may not be included and bias the horizontal inequity indices. In addition, there exist some variations in the definitions of SBA and PNC service utilization variables in terms of the type of health care provider and timings of service provision between the 2000 and 2016 EDHS. Thus, interpretation of findings in this study should consider such variations.

Despite these limitations, this study assessed the objectives using the recent and publicly available data for a relatively long-time period to see the general trends and magnitudes and contributing factors of inequities using the most recent EDHS data that are nationally representative and do have high response rates. The data were collected after high-quality interviewer training was given to the data collectors. The data were collected using a standardized data collection procedures across countries – to make its content consistent over time and comparable across populations [21]. The conclusions of this study were based on the concentration index results, which fulfil the minimum requirement of an inequality measurement rather than inequality ratio results – based on only the extreme quintiles of a population – to compare inequalities across different time periods [18, 19].

Generally, the study shows that the observed increment in the utilization of MHS is only among better off not among poor.

## Conclusion

Our findings show the presence of significantly high and increasing inequities in ANC, SBA, and PNC utilization. The poorer, illiterate, rural residents, not working, and mass media unexposed women (the majority in Ethiopia) were the disadvantaged segments in Ethiopia and health interventions should target them.

## Additional files

Additional file 1: **Figure S1.** ANC service utilization gap trend among poorest and richest population, in Ethiopia from 2000 to 2016. (DOCX 17 kb)

Additional file 2: **Figure S2.** SBA service utilization gap trend among poorest and richest population, in Ethiopia from 2000 to 2016. (DOCX 16 kb)

Additional file 3: **Figure S3.** PNC service utilization gap trend among poorest and richest population, in Ethiopia from 2000 to 2016. (DOCX 16 kb)

## Abbreviations

ADePT: Automated DEC (Development Economics) Poverty Table
ANC: Antenatal Care
CI: Concentration Index/Indices
CONINDEX: Concentration Index
CSA: Central Statistical Agency
EDHS: Ethiopia Demographic Health Survey/s
MHS: Maternal Health Services
PNC: Postnatal care
RCI: Relative Concentration Index/Indices
SBA: Skilled Birth Attendance
WHO: World Health Organization

## Footnotes

1 Maternal mortality is defined as “the death of a woman while pregnant or within 42 days of termination of pregnancy, irrespective of the duration and site of the pregnancy, from any cause related to or aggravated by the pregnancy or its management, but not from the accidental or incidental cause [59].”

2 Inequality in health care is differences in the health care utilization by clusters or person [60, 61]. Whereas, equity in health cares: “is defined as equal access to available care for equal need, equal utilization for equal need, and equal quality of care for all.”
