# Supplementary material for: Inequities in maternal health services utilization in Ethiopia 2000–2016: magnitude, trends, and determinants
Source: Reprod Health. 2018 Jul 4;15:119. doi: 10.1186/s12978-018-0556-x (PMC6031117; doi:10.1186/s12978-018-0556-x)
Supplement: Supplementary file 1 — Figure S1. ANC service utilization gap trend among poorest and richest population, in Ethiopia from 2000 to 2016. (DOCX 17 kb) [file 12978_2018_556_MOESM1_ESM.docx]

**Supplementary material**

**Figure S1**: ANC service utilization gap trend among poorest and richest population, in Ethiopia from 2000 - 2016.
