# Supplementary material for: Inequities in maternal health services utilization in Ethiopia 2000–2016: magnitude, trends, and determinants
Source: Reprod Health. 2018 Jul 4;15:119. doi: 10.1186/s12978-018-0556-x (PMC6031117; doi:10.1186/s12978-018-0556-x)
Supplement: Supplementary file 2 — Figure S2. SBA service utilization gap trend among poorest and richest population, in Ethiopia from 2000 to 2016. (DOCX 16 kb) [file 12978_2018_556_MOESM2_ESM.docx]

**Figure S2**: SBA service utilization gap trend among poorest and richest population, in Ethiopia from 2000 - 2016.
